# Artificial Intelligence in Medical Writing: A Practical and Ethical Framework for Surgical Research and Publication

**DOI:** 10.1002/wjs.70504

**Published:** 2026-07-13

**Authors:** S. R. Thomson, B. A. Bassett, J. E. J. Krige

**Affiliations:** ^1^ Division of Gastroenterology Department of Medicine University of Cape Town Cape Town South Africa; ^2^ MIND Institute and School of Computer Science and Applied Maths University of Witwatersrand South Africa School for Data Science and Computational Thinking Stellenbosch University South Africa Neuroscience Institute University of Cape Town Cape Town South Africa; ^3^ Department of Surgery University of Cape Town and Groote Schuur Hospital Cape Town South Africa

**Keywords:** academic publishing, artificial intelligence, ethics, generative AI, large language models, medical writing, surgical research

## Abstract

**Background:**

Artificial intelligence (AI) is rapidly transforming surgical research and medical publishing by changing how clinicians discover, evaluate, synthesize, and communicate scientific evidence. Despite widespread adoption, practical guidance on the responsible integration of AI into academic writing remains limited, particularly as large language models (LLMs) and emerging AI systems become increasingly sophisticated.

**Methods:**

This narrative review examines the contemporary AI ecosystem relevant to medical writing, including LLMs, retrieval‐augmented systems, structured evidence extraction platforms, citation analytics tools, AI‐enhanced academic databases, and emerging agentic AI systems. Current evidence relating to AI‐assisted manuscript preparation, ethical considerations, governance, confidentiality, reproducibility, and scientific integrity was reviewed. A practical workflow integrating literature discovery, evidence extraction, synthesis, citation validation, and editorial refinement is proposed.

**Results:**

AI can substantially improve the efficiency, organization, clarity, and consistency of manuscript preparation while supporting evidence retrieval, synthesis, and editorial refinement. However, responsible use requires awareness of important limitations, including hallucinated references, publication bias amplification, confidentiality risks, language weighting, and the inability of current LLMs to distinguish reliably between truth and plausibility. The review also highlights the increasing integration of academic databases with AI‐assisted retrieval and synthesis, together with the emergence of agentic research systems capable of performing increasingly complex scientific workflows under human supervision.

**Conclusions:**

AI should be regarded as an adjunct to, not a substitute for, scientific reasoning and scholarly judgment. When used transparently and under expert supervision, AI can enhance the quality and efficiency of medical writing while preserving the central intellectual responsibilities of authorship, interpretation, verification, and accountability. As AI systems become increasingly autonomous, maintaining rigorous human oversight will become progressively more important.

## Graphical abstract

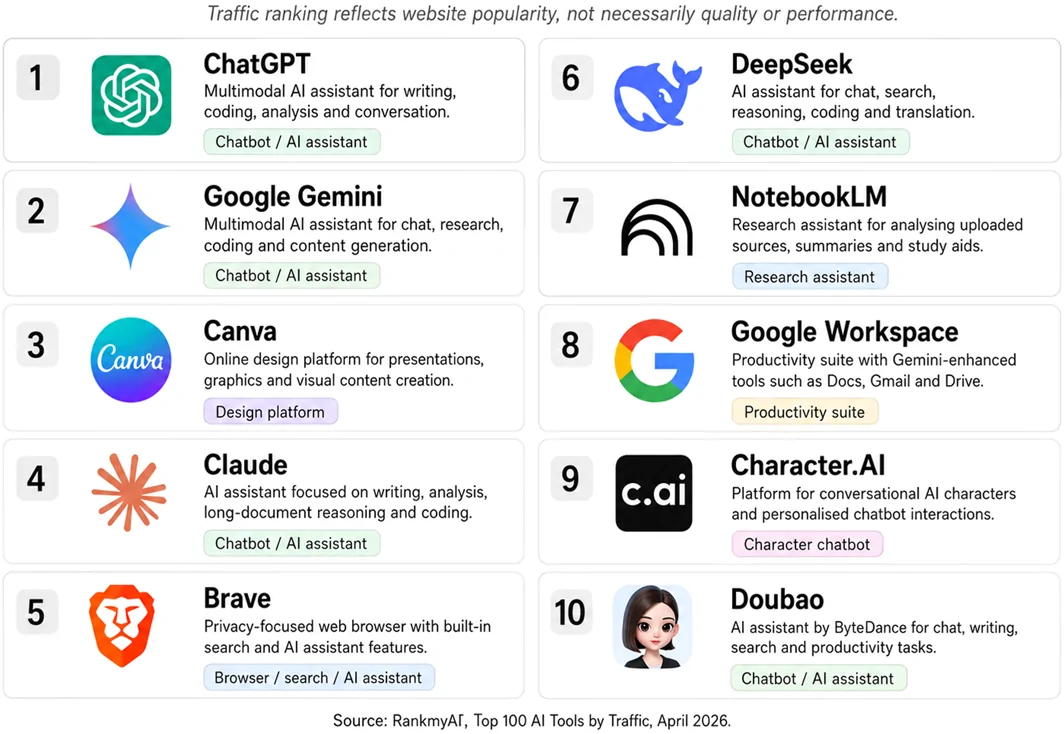

## 1 Introduction

Surgical research is increasingly shaped by technological innovation, yet the integration of artificial intelligence (AI) into academic writing and publication remains incompletely defined. Across surgical disciplines, AI tools are now used for literature discovery, structured evidence extraction, manuscript drafting, editorial refinement, citation analysis, and workflow organization. This transition reflects both technological advancement and increasing pressure on clinician–scientists to produce high‐quality academic output within time‐constrained clinical practice.

Writing in surgery is not merely a linguistic exercise. The construction of an argument, interpretation of results, and contextualization of findings remain central to scientific reasoning. Writing functions not only as a means of communication but as a process through which understanding is developed, tested, and refined [1]. The integration of AI into this process therefore raises a fundamental question: how can efficiency be improved without weakening the intellectual discipline that writing enforces?

The tradition of surgical writing offers an important perspective. Moshe Schein's aphoristic approach to surgical thought emphasizes clarity, brevity, and disciplined reasoning [2]. Embedded within this tradition is recognition that clarity rarely emerges in the first draft. The widely cited principle to “write in haste and edit at leisure” reflects the broader reality that meaning emerges progressively through revision [3]. Revision is not simply stylistic but cognitive; arguments are refined, assumptions challenged, and interpretation strengthened through iterative engagement with text.

AI has the potential to reduce some of the mechanical burden of revision by restructuring sentences, improving flow, refining tone, and organizing information. However, it cannot replace the intellectual work that writing requires. The distinction is crucial. AI may accelerate writing, but it does not independently generate scientific judgment.

These issues are particularly relevant in surgical research, where disparities in mentorship, editorial support, and research infrastructure influence academic participation [4]. AI tools may reduce some barriers related to language, organization, and access to literature synthesis. At the same time, unequal access to advanced systems and variability in their use may create new forms of inequity [5].

While AI is widely discussed within surgery at strategic and clinical levels, the implications for scholarship, writing, and publication remain comparatively underexplored [6]. Given the well‐recognized disparities in global surgical research output, this represents both a challenge and an opportunity [7, 8]. AI offers potential to democratize aspects of academic participation while simultaneously introducing important methodological, ethical, and epistemological concerns.

## 2 The Contemporary AI Ecosystem in Medical Writing

Artificial intelligence in academic writing now extends far beyond individual conversational chatbots. Contemporary systems encompass multiple functional domains including generative language models, retrieval‐augmented search engines, structured extraction platforms, citation analytics tools, workflow assistants, and multimodal image‐generation systems [9, 10, 11, 12].

**FIGURE 1:**
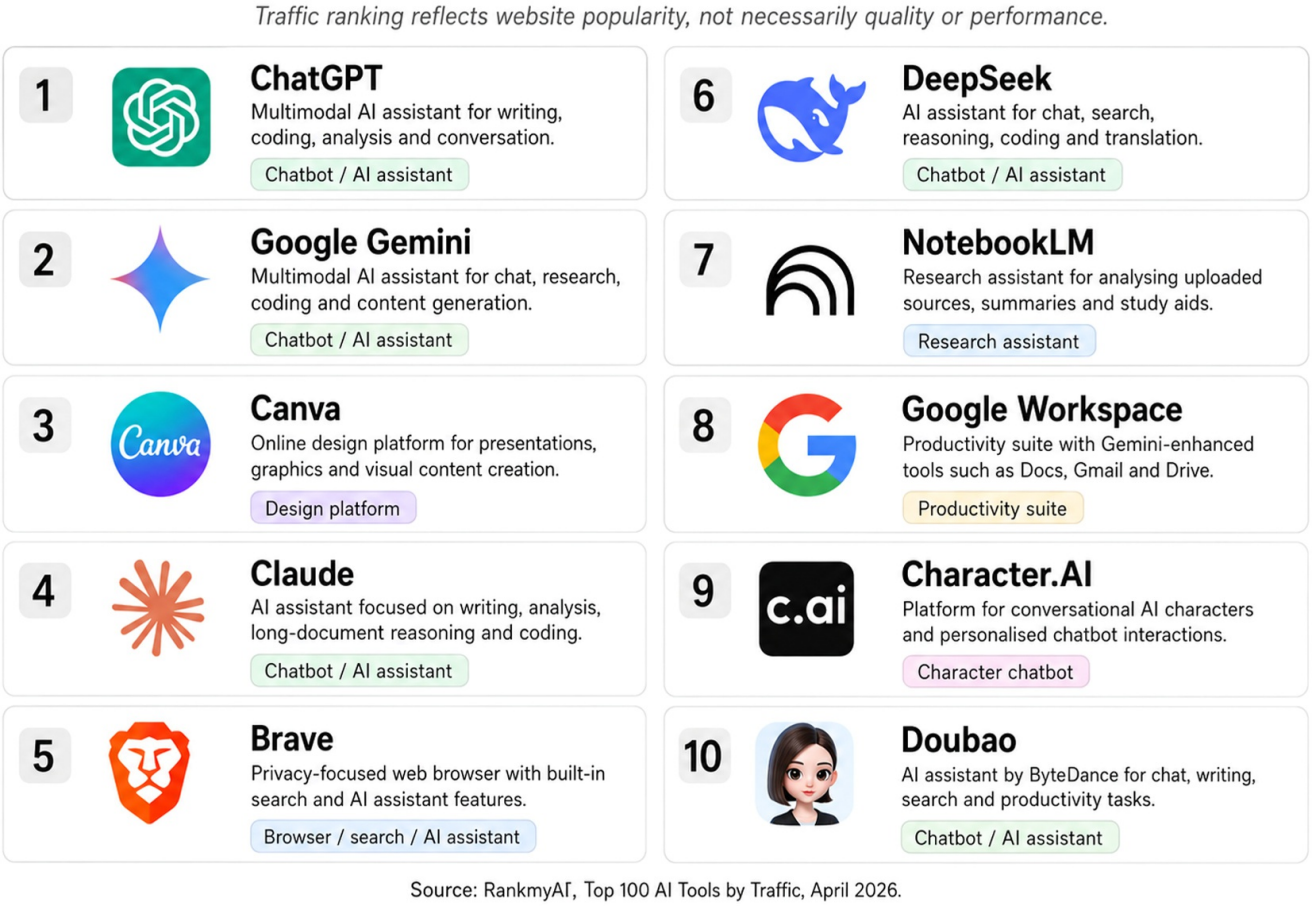
Top 10 artificial intelligence tools by web traffic.

Figure 1 shows the characteristics of the 10 most used AI applications by global usage as of April 2026, derived from traffic data reported by RankMyAI [13].

These applications are widely used across industries for writing, research, productivity, creativity and problem solving.

Although ChatGPT has become the most visible generative AI platform within medicine, it represents only one component of a rapidly expanding ecosystem. Other systems including Claude, Gemini, Microsoft Copilot, Perplexity AI, Grammarly, Notion AI, Midjourney, and Omni provide complementary capabilities involving retrieval augmentation, editorial refinement, workflow integration, image and video generation, and citation analysis.

No single AI platform currently reproduces the complete intellectual workflow of scientific research and publication, though the gaps are closing rapidly. Effective AI‐assisted writing therefore still benefits from combining systems with complementary strengths under human oversight.

The contemporary AI landscape can be conceptualized according to functional roles rather than individual brands. Large language Models (LLMs) primarily support idea generation, synthesis and drafting, retrieval‐augmented systems improve evidence retrieval and source linkage, structured extraction tools facilitate systematic organization of evidence, and citation analytics platforms enhance contextual validation of claims.

This fragmentation remains important because current AI systems generally augment specific components of academic work rather than replacing the broader intellectual processes of scientific reasoning, interpretation, and judgment. Nevertheless, the pace of development is rapid. Recent advances include AI systems capable of solving previously intractable mathematical problems [14] and the emergence of multi‐agent AI co‐scientists [15], in which multiple specialized AI agents collaborate on tasks such as literature review, hypothesis generation, experimental design, analysis, and critique under the coordination of a supervisory agent. These systems can independently execute substantial components of the scientific method, suggesting that the boundary between assistance and autonomy is becoming increasingly fluid. As AI capabilities expand, the need for human oversight, verification, and accountability becomes greater rather than less.

A related consideration is data governance and confidentiality. The risks associated with AI platforms depend not only on the provider but also on the subscription model used (Table 1). Individual consumer subscriptions generally offer fewer governance controls than institutional licenses or managed API services designed for universities, hospitals, and research organizations. Such institutional arrangements may include contractual data‐protection agreements, administrative oversight, audit capabilities, tenant isolation, and restrictions on the use of submitted content for future model training. Consequently, the appropriateness of uploading unpublished manuscripts, reviewer comments, research protocols, or patient‐related information should be assessed according to the specific platform and subscription model rather than the AI system alone.

**TABLE 1:** Privacy protections and confidentiality risks of commonly used AI platforms for academic writing according to subscription type.

Platform	Consumer/individual paid version	Institutional version available	Typical risk for unpublished academic data*
ChatGPT	User can usually opt out of training, but data still resides within OpenAI systems for 90 days	Yes	Moderate (individual)/low–moderate (institutional)
Claude	Generally strong privacy protections, but not designed specifically for institutional governance	Yes	Moderate (individual)/low–moderate (institutional)
Microsoft copilot	Consumer version separate from organizational environment	Yes	Moderate (consumer)/low (institutional)
Gemini	Consumer and workspace versions differ substantially	Yes	Moderate (consumer)/low–Moderate (institutional)
Notion AI	Depends on third‐party model integrations	Limited	Moderate
Grammarly	Cloud processing required	Yes	Moderate
Perplexity	Policies evolving	Limited	Moderate
Jasper	Commercial/business focus	Yes	Moderate
Midjourney	Public‐generation culture and limited confidentiality focus	No meaningful institutional model	Higher
DALL·E	Not designed for confidential research content	Limited	Moderate–higher

*Note:* Comprehensive: Formal organizational controls, contractual privacy agreements, administrative oversight, and restrictions on model training. Moderate: Some organizational controls available but less comprehensive governance. Limited: Basic privacy features present but few dedicated institutional governance mechanisms. Minimal: Primarily consumer‐focused platform with limited confidentiality protections.

## 3 Capability and Access: Free Versus Advanced Systems

A further distinction exists between free‐tier and advanced AI systems. Free tools typically provide access to smaller, cheaper and faster models supporting more basic functionality including editorial, grammar correction, summarization, and basic restructuring. These functions are valuable, particularly for improving clarity and accessibility, but do not provide deep analytical capability.

Costly advanced systems, based on larger models allowed to think longer, enable more sophisticated reasoning, larger context handling, and deeper synthesis. This distinction becomes particularly apparent in discussion writing, where integration of heterogeneous evidence and critical interpretation are required and in coding and solving mathematical challenges.

However, increased capability does not necessarily equate to reliability, though there has been rapid progress on all relevant benchmark evaluations and independent model leaderboards [16]. Even advanced systems benefit from careful prompting, verification, and oversight. The difference between superficial and expert use lies not only in access to technology but in the manner in which it is applied.

A detailed comparison of platform‐specific privacy policies, retention practices, and governance provisions is provided in Table S1.

## 4 How Large Language Models Generate Language

What sits behind a large language model can sound forbiddingly technical—tokens, vector spaces, transformer layers. Despite the technical complexity, the underlying principles are less alien than they first appear. Large language models construct language through highly sophisticated statistical prediction [8, 17]. LLMs first fragment language into smaller units known as tokens, representing words, word fragments, punctuation, or syntactic elements. These are converted into mathematical representations known as vectors. Much of the power of modern AI derives from the ability to represent language, images, and audio within a shared mathematical space, where semantic similarity is reflected by proximity between vectors. Words, sentences, and paragraphs with related meanings therefore occupy neighboring regions of this representation space.

The next key innovation, implemented through transformer architectures and attention mechanisms, enables LLMs to identify which parts of the preceding text are most relevant to predicting what comes next [18]. Thus, in “the fisherman sat on the bank …” and “the customer entered the bank …” attention mechanisms infer different meanings for *bank* based on context. This markedly improves language generation. Predicting the next word remains the fundamental capability of base LLMs [17], but modern systems extend this through reinforcement learning and preference optimization, training models to generate responses that humans are more likely to prefer. This breakthrough underpinned the original success of ChatGPT. However, aligning outputs with predicted human preferences may also contribute to sycophancy and the avoidance of conclusions likely to be perceived as unpopular or uncomfortable. Since 2024, LLMs have been further enhanced through reasoning architectures that allow models to generate intermediate deliberative steps during inference. Rather than producing immediate answers, these systems can decompose problems, explore alternative solution paths, revise errors, and in some cases use external tools before returning a response. Higher levels of reasoning generally require greater computational resources and longer response times.

Despite these advances, LLMs do not currently possess a reliable causal ability to distinguish truth from plausibility outside domains where formal verification is possible. Their outputs remain grounded primarily in learned statistical relationships rather than verified understanding. The resulting prose may therefore appear balanced, coherent, and authoritative while lacking genuine conceptual comprehension [5, 10].

In academic writing, this creates an important epistemic challenge. The boundary between assistance and authorship becomes increasingly difficult to define as AI‐generated text acquires persuasive fluency. A paragraph may appear rigorous while acquiring an appearance of authority through statistical resemblance to human scholarly discourse. The significance of AI in medical writing therefore lies not simply in automation, but in the growing convergence between meaning and mimicry. The ethical and epistemological questions surrounding AI‐assisted scholarship arise from this convergence [8, 19, 20].

## 5 Categories of AI Tools and Their Functions

AI tools used in medical writing operate across several interrelated domains including discovery, extraction, evidence scanning, synthesis, validation, and editorial refinement. Understanding these domains is essential for their responsible use.

No single platform replicates the full intellectual process of medical research. An important limitation across these domains is fragmentation: outputs are not inherently integrated, and the responsibility for synthesis remains with the author. Furthermore, AI outputs may reflect underlying publication and indexing biases [10]. The absence of evidence within AI outputs should not be interpreted as evidence of absence.

## 6 Practical Optimization of Large Language Models in Medical Writing

LLMs represent the most transformative component of the current landscape because they bridge language and reasoning. Widespread adoption reflects the ability of LLMs to generate structured, coherent text; however, their primary value lies in analysis rather than editing [21]. When appropriately directed, these systems can synthesize evidence, reconcile conflicting findings, and contextualize results within clinical practice, functioning as analytical tools rather than simple writing aids [21]. Effective use depends on structured prompting, iterative refinement, and anchoring to source material. The distinction between superficial and expert use is most evident in prompting strategy, which strongly influences output quality [21]. A generic instruction such as “summarize this paper” typically yields descriptive output with limited analytical depth, whereas structured prompts, requesting comparison of methodology, identification of bias, and interpretation of clinical implications, produce more substantive responses. Task segmentation further improves output quality. Rather than generating entire manuscript sections in a single step, higher‐quality results are achieved by dividing tasks into discrete components, such as synthesis, critical appraisal, and stylistic refinement. Iterative refinement remains central, enabling rapid cycling through drafts and progressive improvement in clarity, coherence, and analytical depth. This reflects traditional writing processes, in which clarity emerges through repeated revision [3], while substantially reducing the time required for each iteration. Anchoring outputs to source material improves reliability and reduces unsupported claims [12]. Structured inputs enhance precision, whereas unstructured prompts may dilute accuracy. LLMs are also effective in managing revision under word‐count constraints, enabling progressive condensation of complex arguments without disproportionate loss of meaning.

These principles align with established traditions of academic writing. AI accelerates the iterative process of revision, but this efficiency applies primarily to the mechanics of writing rather than to reasoning itself. As the effort required to produce fluent text decreases, there is a risk that critical reflection may be abbreviated. The fluency of AI‐generated prose can create an illusion of completeness while underlying reasoning remains underdeveloped [1]. Maintaining revision as a cognitive process, rather than a purely editorial one, is therefore essential.

## 7 The Role of Academic Databases in the AI Era

Traditional academic databases remain essential for high‐integrity research, functioning as structured evidence repositories increasingly augmented by AI‐assisted search and citation tools. Historically, a clear distinction existed between databases, which stored and organized evidence, and AI systems, which generated interpretations. That distinction is becoming less clear. Contemporary platforms increasingly combine retrieval, citation analysis, evidence extraction, semantic search, and AI‐assisted synthesis within a single environment.

Despite this convergence, the underlying functions remain distinct. Academic databases continue to provide the traceable, source‐linked evidence upon which scholarly interpretation depends, whereas generative systems assist in organizing, contextualizing, and synthesizing that evidence. High‐quality medical writing increasingly relies on the integration of both capabilities within a unified workflow rather than on either approach alone (Table 2).

**TABLE 2:** Functional roles of AI‐assisted tools in academic and medical writing.

Tool	Primary function	Key strength	Limitation	Best use
Research rabbit	Discovery	Citation mapping, trend identification	No synthesis	Early literature exploration
Scite	Citation validation	Evidence verification	No drafting capability	Strengthening arguments
Consensus	Evidence scanning	Evidence‐grounded summaries	Limited depth	Checking clinical claims
Elicit	Structured extraction	PICO‐based organization	Limited narrative synthesis	Systematic reviews
LLMs	Synthesis & drafting	Deep analytical integration	Requires citation verification	Discussion writing, manuscript drafting
“Deep research” mode on OpenAI, gemini and perplexity	Discovery and evidence synthesis	Citation mapping, trend identification and deep integration	Blackbox search criteria	Broad literature exploration and synthesis

*Note:* Structured extraction tools support the organization of study characteristics, outcomes, and methodologies into tabulated formats, particularly in systematic reviews where transparency is essential [10]. Evidence‐scanning tools provide rapid summaries of findings across the literature, but may lack depth in methodological critique [11]. LLMs occupy a distinct role, enabling synthesis and narrative construction as a complementary analytical tool, while also introducing risks such as hallucinated citations and overgeneralization [12]. Citation validation tools analyze citation context, supporting balanced interpretation and reducing selective citation bias [12]. These tools taken together, these tools serve distinct but interrelated roles.

**FIGURE 2:**
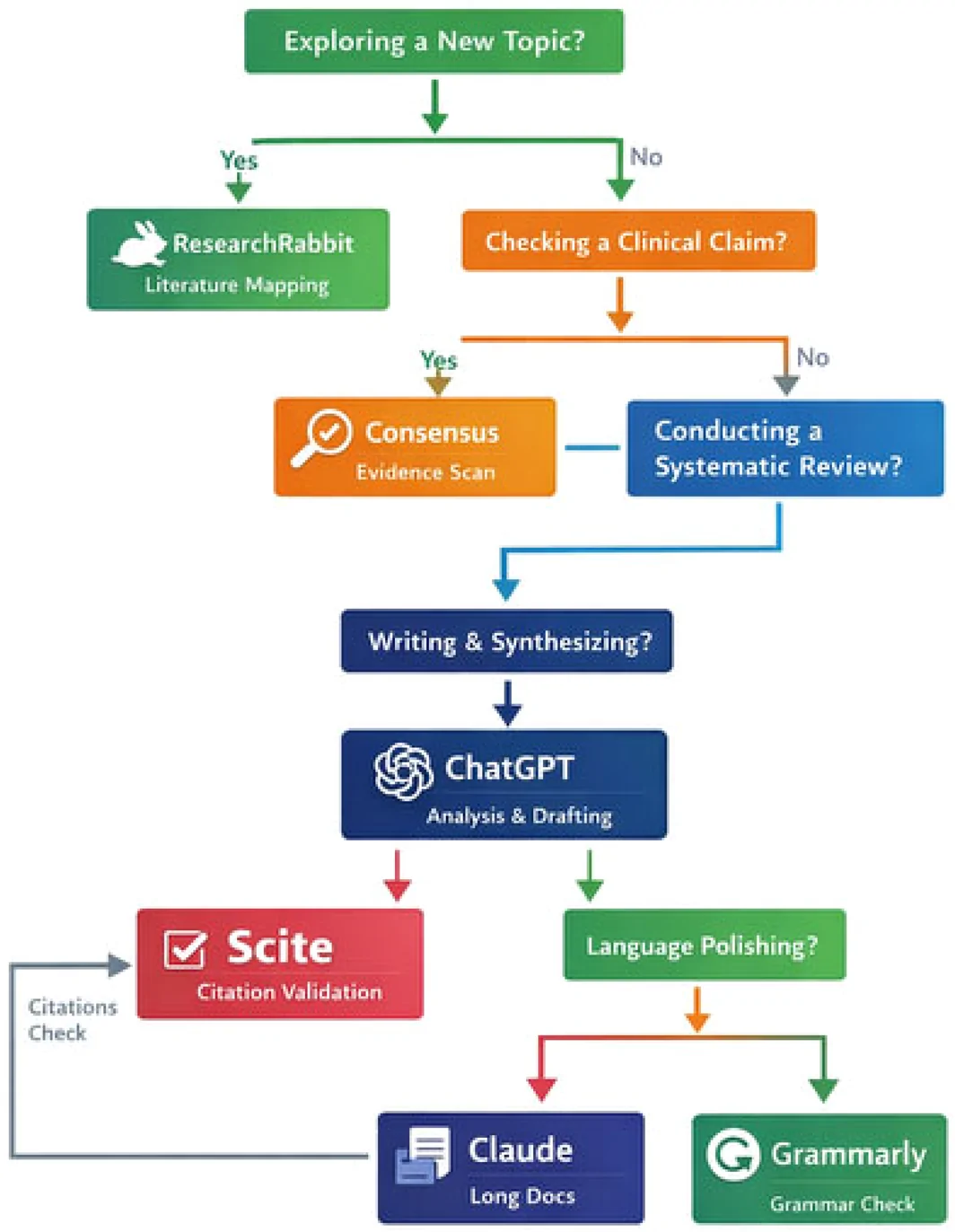
The artificial intelligence assisted surgical writing framework.

The integration of AI into surgical scholarship can therefore be viewed as the evolution of a continuum rather than a sequence of separate tools (Figure 2).

Discovery, extraction, synthesis, and validation are increasingly interconnected processes, with modern platforms combining functions that were previously performed independently. This trend is likely to accelerate further with the emergence of agentic AI systems, in which multiple specialized agents perform retrieval, appraisal, synthesis, and revision under the coordination of a supervisory agent [20]. As research workflows become increasingly integrated and semi‐autonomous, the importance of human oversight, verification, and accountability becomes greater rather than less [20].

## 8 Ethical Considerations

The integration of AI into medical writing introduces important ethical considerations. Authorship remains a human responsibility, and AI cannot be credited as an author [22, 23, 24]. Transparency is increasingly emphasized, with a growing number of journals requiring disclosure of AI use [25]. However, current guidance remains heterogeneous and often fails to specify how AI has been used in manuscript development [25, 26].

Meaningful transparency requires more than generic statements of AI use; disclosures should identify the tools employed and their specific role within the writing process. Whether this improves editorial assessment depends on how such information is structured, simply listing tools may add little, whereas clarifying how AI contributed to literature synthesis, drafting, or revision may allow more informed appraisal of methodological and interpretive integrity [27]. Accuracy remains a central concern. LLMs may produce fabricated or unsupported references, necessitating independent verification [12]. In addition, AI systems may reflect underlying biases within the literature, including publication bias and the overrepresentation of high‐impact sources [27].

Additional concerns include authorship integrity [23, 24], risks to scientific transparency [25], performance variability in healthcare contexts [28], implications for clinical workflows [29], examination performance [30], and the broader need to define responsible AI integration in science [20]. However at the same time, AI has the potential to democratize academic writing by reducing barriers related to language proficiency and access to editorial support, particularly in resource‐limited settings. Beyond these issues lies a more subtle epistemic risk: the fluency of AI‐generated text may create an illusion of understanding [26]. Well‐structured prose can obscure gaps in reasoning, leading to overconfidence in conclusions that have not been critically interrogated, both for the author and the reader. Regardless of the degree of AI assistance, responsibility for accuracy, interpretation, and scientific integrity remains entirely with the human author.

## 9 Assistance Versus Substitution

A critical distinction must be maintained between AI as assistance and AI as substitution. Writing is fundamentally a process of reasoning rather than mere communication. Drafting and revision expose gaps in understanding, challenge assumptions, and refine interpretation. AI can improve efficiency, organization, and clarity, but cannot substitute for the intellectual work required to develop and defend scientific arguments [31].

The risk is not that AI will think for authors, but that increasingly fluent text may create the impression that sufficient human thinking has already occurred. Responsible use therefore requires ensuring that text has been exposed to sufficient intellectual rigor.

## 10 Conclusion

Artificial intelligence is now an established component of surgical writing, with recognized benefits across literature discovery, evidence synthesis, manuscript preparation, and editorial refinement.

In resource‐limited settings, AI may reduce barriers related to language proficiency, access to literature, and editorial support. However, these advantages depend on critical oversight; uncritical reliance risks reinforcing superficial reasoning, amplifying existing biases, and weakening interpretive rigor.

The central challenge is therefore not whether AI can generate convincing text, but how such systems can be integrated responsibly into the discipline of critically interrogating, refining, and communicating ideas. As AI evolves from tools that generate text to systems capable of independently performing increasingly complex tasks, the need for human judgment, verification, and accountability will become even more important. Whatever these tools become, the duty to interrogate their output critically will only grow. The tools are new; the intellectual responsibilities remain unchanged.

## Author Contributions

**S. R. Thomson:** conceptualization, methodology, writing – original draft, writing – review and editing, validation. **B. A. Bassett:** methodology, writing – review and editing. **J. E. J. Krige:** conceptualization, methodology, validation, writing – review and editing.

## Funding

The authors have nothing to report.

## Conflicts of Interest

The authors declare no conflicts of interest.

## Supporting information


**Table S1:** Privacy protections and confidentiality risks of commonly used AI platforms for academic writing according to subscription type.

## Data Availability

Data sharing not applicable to this article as no datasets were generated or analyzed during the current study.
