# Supplementary material for: Artificial Intelligence in Medical Writing: A Practical and Ethical Framework for Surgical Research and Publication
Source: World J Surg. 2026 Jul 13;50(9):2625–32. doi: 10.1002/wjs.70504 (PMC13576536; doi:10.1002/wjs.70504)
Supplement: Supplementary file 1 [file WJS-50-2625-s001.docx]

| **Supplementary Table.**  Privacy protections and confidentiality risks of commonly used AI platforms for academic writing according to subscription type | | | |
| --- | --- | --- | --- |
| **Platform** | **Consumer / Individual Paid Version** | **Institutional Version Available** | **Typical Risk for Unpublished Academic Data*** |
| ChatGPT (OpenAI) | Personal ChatGPT Free/Plus/Pro: data sharing is generally enabled by default, but users can opt out for future chats. Chats are saved until deleted; deleted chats and Temporary Chats are normally removed from OpenAI systems within 30 days, subject to legal/security exceptions. | Yes. ChatGPT Business, Enterprise, Edu and API offerings. Inputs and outputs are not used for training by default. Enterprise/workspace retention, security and admin controls are available. | Moderate for individual accounts; Low–Moderate for institutional accounts. |
| Claude (Anthropic) | Claude Free/Pro/Max are consumer services. Anthropic states that consumer chats/coding sessions are used to improve Claude only if the user allows this, but consumer accounts still lack institutional governance controls. | Yes. Claude Team, Enterprise and API. Commercial inputs and outputs are not used for model training by default; enterprise retention and admin controls are available. | Moderate for individual accounts; Low–Moderate for institutional accounts. |
| Microsoft Copilot | Consumer Copilot/Copilot Pro is separate from an organisation’s Microsoft 365 tenant and should not be treated as an institutional research environment. | Yes. Microsoft 365 Copilot and Copilot Chat with enterprise data protection. Prompts, responses and Microsoft Graph data are not used to train foundation LLMs; interactions are logged/stored in the tenant for governance, retention and eDiscovery. | Moderate for consumer use; Low for managed institutional use. |
| Gemini (Google) | Consumer Gemini Apps can use activity to improve Google services, including training generative AI models, depending on settings. Default auto-delete is 18 months; some reviewed data may be retained longer. Google explicitly warns users not to enter confidential information they would not want reviewers to see. | Yes. Gemini in Google Workspace. Workspace chats and uploaded files are not reviewed by humans or used to train generative AI models without permission, and are governed by the organisation’s Workspace agreement and data-processing terms. | Moderate–Higher for consumer use; Low–Moderate for Workspace use. Consumer Gemini is not appropriate for confidential unpublished academic data. |
| Notion AI | Notion AI is embedded in the Notion workspace and may use workspace pages, connected apps and web search depending on settings. Notion states that customer data is not used to train models by default; non-Enterprise LLM-provider retention is generally 30 days or fewer. | Yes. Notion Enterprise. LLM providers use zero data retention by default for Enterprise workspaces; enterprise controls include integrations and security/compliance features. | Moderate for non-Enterprise workspaces; Low–Moderate for Enterprise. Risk increases if broad workspace or connector access exposes more unpublished material than intended. |
| Grammarly | Grammarly requires cloud processing of text. Users/admins should check the Product Improvement and Training control; saved documents may remain until deleted. | Yes. Grammarly Enterprise, Business and Education. For Enterprise-tier and Education accounts, Product Improvement and Training is off by default; enterprise encryption/key-management options are available. | Moderate for individual use; Low–Moderate for institutional use. Suitable for editing lower-risk unpublished text if institutional settings are verified; avoid sensitive datasets or identifiable material. |
| Perplexity | Free/Pro/Max accounts have AI Data Retention enabled by default, with an opt-out for future data. Some data may be used for model training/search-quality improvement unless disabled. | Yes. Perplexity Enterprise/Enterprise Max. Enterprise data is not used for training; uploaded-file retention and admin controls are available; third-party model providers are contractually prohibited from training on Perplexity data. | Moderate for individual use; Low–Moderate for Enterprise. Avoid confidential unpublished research in consumer mode, especially uploaded documents. |
| Jasper | Jasper is primarily a marketing/business content platform. Creator/Pro and Business contractual terms differ, and the product is not primarily designed as an academic research-data environment. | Yes. Jasper Business/enterprise under SaaS/DPA terms. Jasper’s SaaS agreement states that Customer Property is not used, and third parties are not permitted to use it, to train AI models used by the service. | Moderate for individual/creator use; Low–Moderate for Business/enterprise use with an appropriate contract. |
| Midjourney | Image-generation service with a public-generation culture. Stealth Mode is available only on Pro and Mega plans, and does not hide creations made in public Discord channels. | Limited. No widely comparable institutional governance model to Microsoft 365 Copilot, OpenAI Enterprise, Google Workspace Gemini or Claude Enterprise. | Higher. Do not use for confidential unpublished academic data, identifiable research participants, confidential diagrams, unpublished visual results or patentable visual concepts unless privacy terms are specifically contracted and settings are verified. |
| OpenAI image generation / DALL·E | Best treated as part of the OpenAI account used rather than as a separate governance category. In personal ChatGPT, the same personal data controls and retention considerations apply. Image prompts/uploads may themselves contain confidential IP even if outputs are not public by default. | Yes, through ChatGPT Business/Enterprise/Edu and the OpenAI API. Business/API data is not used for training by default and retention controls may apply. | Moderate for individual use; Low–Moderate for institutional/API use. Safer than Midjourney for confidentiality only when used under approved OpenAI business/API terms. |

* Risk categories are indicative, not legal advice or a security certification. They assume ordinary unpublished academic material. Identifiable human-subject data, health records, student records, grant-review material, embargoed manuscripts, patentable inventions, third-party confidential data or regulated data should be used only in institutionally approved tools with appropriate contractual, retention, access-control and data-processing safeguards.
